# Molecular mimicry and trafficking of peptide effectors in sedentary nematodes: emerging drivers of feeding site formation and host signaling hijack

**DOI:** 10.1007/s44297-025-00063-2

**Published:** 2026-01-06

**Authors:** Abdulmujib Gboyega Yusuf, Tesleem Taye Bello, Saheed O. Anifiwoshe

**Affiliations:** 1 Plant Protection Department, College of Food and Agricultural Sciences, King Saud University, P.O. Box 2460, 11451 Riyadh, Saudi Arabia; 2 Department of Agricultural Science Education, Federal College of Education, Ogun State, PMB 2096, Abeokuta, Nigeria; 3 Zoology Department, College of Science, King Saud University, P.O. Box 2455, 11451 Riyadh, Saudi Arabia

**Keywords:** Plant-parasitic nematode effector, Hormone mimicry, Immune suppression, Feeding site formation, Plant-nematode interaction

## Abstract

Despite significant advances in understanding the biology of plant-parasitic nematodes, the emergence of peptide hormone mimicry as a virulence strategy presents a complex facet of nematode parasitism. This review integrates recent advances on how nematode effectors, such as CLEs, CEPs, RALFs, IDA, and PSYs, are processed, post-translationally modified, and trafficked to hijack host signaling and developmental programs. By linking structural mimicry with receptor engagement and subcellular targeting, we highlight how these effectors reprogram plant transcriptional and immune responses to drive the formation of nematode feeding sites. We further explore the evolutionary origins of these effectors, emphasizing how processes such as horizontal gene transfer, neofunctionalization, and convergent selection have shaped peptide effectors into lineage-specific virulence factors. Finally, we outline critical research gaps focusing on structural and computational analyses of effector-receptor interfaces, functional genomics of trafficking and activation and translational opportunities for engineering durable host resistance. Together, these insights underscore the influence of molecular mimicry on nematode virulence and position effector biology as a frontier for translational innovation in crop protection.

## Introduction

Plant-parasitic nematodes (PPNs) are important crop disease-causing pathogens, constituting a major constraint to global agricultural productivity. These species are known to infect a wide range of major crops, with annual crop losses exceeding $170 billion [1, 2]. Among them, sedentary endoparasitic species, such as root-knot nematodes (RKN; *Meloidogyne* spp.) and cyst nematodes (CN; *Heterodera* and *Globodera* spp.), cause the greatest damage due to their ability to establish long-term feeding relationships within host plant roots [3]. Unlike migratory species, sedentary PPNs induce the formation of permanent feeding sites, including giant cells or syncytia, by profoundly reprogramming the development and metabolism of host cells [4]. From the feeding site, the nematode obtains the required nutrients and water, which can significantly impact the host's development or even lead to host death in some cases. This biotrophic interaction is sustained through the secretion of effector proteins. Effectors are pathogen-derived molecules that modify host cellular processes and functions to the advantage of the parasitizing pathogen [5]. Crucially, in PPNs, effectors are the key virulence molecules produced in the nematode's esophageal glands and are injected directly into host cells via a stylet [6].

Evidence-based studies strongly suggest that effector proteins are crucial for nematode parasitism [7, 8]. The nematodes engage effector proteins to manipulate selected root cells into a specialized, permanent nematode feeding site (NFS). Without NFS, successful parasitism cannot occur, aborting the nematode life cycle abruptly or leading to the emergence of several males that can gain mobility and move out of the host tissue [9, 10]. Notably, the effectors achieve this by facilitating host penetration, suppressing plant immune responses, remodeling cell walls, and hijacking plant signaling pathways to redirect host developmental processes, which underscores the multifaceted nature of their roles in the infection process. In essence, fully understanding the mechanism of nematode parasitism necessitates a thorough characterization of their effectors. In addition, identifying their host target and understanding their functions are crucial steps in this endeavor [11, 12]. These effectors are generally classified by their diverse biological roles, including immune suppression, cell wall modification, hormone mimicry, and induction of feeding sites. However, despite notable progress in effector identification through genomics and transcriptomics, the functional characterization of many effector proteins, especially those lacking homology to known protein families, remains a significant challenge.

Among the most intriguing strategies employed by sedentary nematodes is molecular mimicry, resulting from the evolution of effector proteins that structurally and functionally resemble host molecules [13]. Through this strategy, nematodes hijack plant signaling networks to promote parasitism. Some effectors mimic endogenous plant peptides, such as CLAVATA3/EMBRYO SURROUNDING REGION-related (CLE), C-terminally encoded peptides (CEPs), rapid alkalinization factors (RALFs), inflorescence deficient in abscission (IDA) proteins, and plant peptides containing sulfated tyrosine (PSY) [14, 15]. Other mimic enzymatic or structural host proteins, such as chorismate mutases or annexin-like proteins [16], further illustrate the diverse routes through which molecular mimicry enhances virulence.

Peptide-mimicking effectors, in particular, are capable of engaging host receptor kinases and modulating developmental processes, including vascular patterning, organ separation, and root meristem activity. Ultimately, some effectors interfere with host transcription factors and hormonal networks, mitigating pattern-triggered and effector-triggered immunity [17, 18]. Deciphering how these effectors are processed in planta, post-translationally modified, trafficked, and delivered to their precise subcellular locations remains critical to understanding their role in host manipulation.

While recent genomic resources have greatly expanded the known repertoire of nematode effectors, key questions persist. Functional redundancy, dual localization patterns, and the need for host-like post-translational modifications further complicate effector studies [19–21]. Moreover, the evolutionary origin of these effectors, whether arising via horizontal gene transfer, gene duplication, or neofunctionalization, is yet to be completely understood. Therefore, a comprehensive synthesis specifically examining the intertwined role of molecular mimicry and intracellular trafficking of peptide effectors in sedentary nematode parasitism, particularly concerning NFS formation and host signaling hijack, remains underexplored. Therefore, this review focuses on a unique subset of nematode effectors, peptide hormone-like molecules mimicking host signaling peptides, with critical roles in feeding site formation and developmental reprogramming. We explore how these effectors are synthesized, modified, and delivered, alongside their engagement with host receptors, and how their evolution has shaped parasitism in sedentary nematodes. By integrating recent advances in effector biology with insights into molecular mimicry and host signaling hijack, we provide a conceptual framework for understanding nematode virulence and identify new avenues for effector-targeted nematode control strategies.

### Biology and infection strategy of sedentary nematodes

Sedentary endoparasitic nematodes, such as RKN and CN, establish long-term feeding relationships with their host plants through a highly specialized parasitic lifestyle. Their life cycle begins with an egg, within which the first-stage juvenile (J1) develops and undergoes its first molt to become a second-stage juvenile (J2). Upon hatching, J2 is stimulated and attracted to host roots and penetrates the root epidermis mechanically using its stylet and enzymatically by secreting cell wall-degrading enzymes to facilitate its penetration and migration [22, 23]. This initial invasion is critical for accessing host tissues and initiating feeding site formation before the nematode becomes permanently sedentary and completely relies on its host for survival.

Root-knot nematodes typically migrate intercellularly and trigger the transformation of several parenchyma cells into hypertrophied, multinucleated giant cells (GCs) through repeated nuclear division without cytokinesis [24, 25]. In contrast, CNs follow a different route, migrating intracellularly through the root cortex while dissolving cell walls [26, 27]. Upon reaching the vascular cylinder, they select an initial feeding cell where they induce the fusion of adjacent cell protoplasts to form a large multinucleated feeding structure known as the syncytium [28]. Despite differing developmental pathways, both GCs and syncytia exhibit elevated metabolic activity and serve as essential nutrient sources throughout the nematode's sedentary phase.

The formation and maintenance of these feeding sites involve extensive reprogramming of host cellular architecture, transcription activity, and defense signaling. These changes are largely orchestrated by effector proteins, specialized molecules secreted by the nematode and delivered into plant cells via stylets. Among these, a notable group of effectors structurally and functionally mimics host peptide hormones, enabling nematodes to co-opt plant developmental programmes. These include CLE-like peptides influencing meristem activity, CEP-like peptides modulating nutrient signaling, IDA-like peptides promoting cell separation, RALF-like peptides targeting stress and immune responses, and PSY-like peptides mediating host signaling [29–33] Table 1. These mimicry strategies reflect the evolutionary sophistication of sedentary nematodes and highlight the importance of understanding not only effector function but also their secretion dynamics and intracellular trafficking during infection. To fully unravel how these molecules operate within host tissues, it is essential to examine where and when they are produced, how they are processed, and the mechanisms by which they are delivered to specific cellular compartments.

**Table 1:** A comparative overview of the functional features of the major peptide mimicry effector families

Effector family	PPN mimicry effector gene	Presence of Pro-domain	Known PTMs	Host Receptor	Primary host-process manipulated	References
CLE	*HgCLE1* *MiCLE*	Present in CN; Absent in RKN	Proline Hydroxylation, Arabinosylation	CLAVATA1 (CLV1), TRACHEARY ELEMENT DIFFERENTIATION FACTOR RECEPTOR (TDR)	Meristem maintenance, vascular patterning, and cell proliferation	[49, 51, 59, 66, 67]
CEP	*MhCEP1*	Absent in RKN	Hydroxylation	CEP RECEPTOR1 (CEPR1)	Root elongation, nitrogen signaling, and modulation of lateral root growth	[71, 72]
IDA	*MilDL1* *MilIDL2*	Absent	Hydroxylation	HAESA (HAE), HAESA-LIKE2 (HSL2)	Cell separation and wall remodeling during NFS formation	[31]
PSY	*MigPSY1-3*	Short/Absent	Tyrosine sulfation	PSY-LIKE RECEPTOR HOMOLOGS (e.g., AtPSY1R)	Cell expansion, gall hypertrophy, and developmental reprogramming	[32]
RALF	*MiRALF1* *MiRALF3*	A short form is present in RKN	Not confirmed	FERONIA (FER)	Cell wall loosening, modulation of immune signal	[82, 93]

### Secretion pathways and effector delivery in sedentary nematodes

The ability of sedentary plant-parasitic nematodes to manipulate host cellular processes depends on the precise and timely secretion of effector proteins [10, 34]. These molecules are produced in specialized esophageal gland cells, mainly the subventral glands (SvGs) and a dorsal gland (DG), and are delivered through the stylet. This hollow, retractable structure injects effectors directly into host tissues. Most effector secretion follows the standard endoplasmic reticulum (ER)-Golgi pathway [35]. However, some effectors have been localized to other secretory structures, such as the amphid (Mh-TTL2) and the nematode cuticle (Mi-MIF2), indicating the presence of alternative pathways for effector development [36, 37].

During early infection, SvGs are predominantly active and produce effectors that facilitate tissue penetration, intercellular migration, and evasion of early plant immune responses [38, 39]. The plant cell wall presents a significant barrier, particularly during the invasive activity of the infective J2 stage. Root-knot nematodes, for instance, must weaken the boundaries between cells to enable their penetration and subsequent intercellular migration through root tissue. This process is largely aided by the secretion of various CWDEs, including pectate lyases, β−1,4-endoglucanases, and xylanases [40, 41]. Notably, genomic analysis of *Meloidogyne incognita* revealed over 81 CWDES-encoding genes [42]. In addition to CWDEs, early-stage effectors include detoxification proteins and immune suppressors that dampen plant responses during invasion [43].

**Fig. 1:**
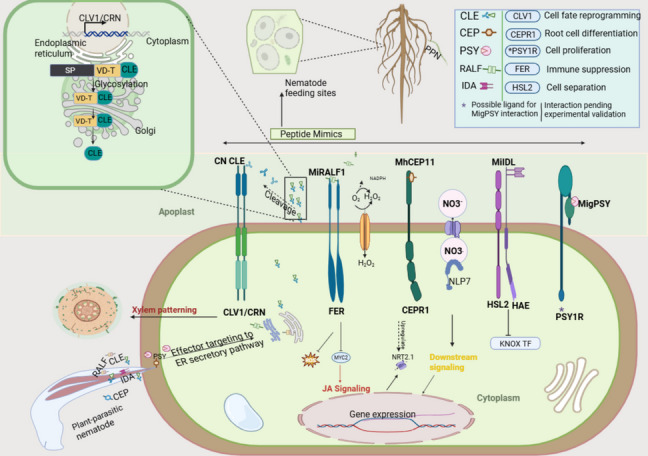
Peptide Mimicry Effectors and Their Functional Targets in Plant Host Cells. Sedentary plant-parasitic nematodes secrete a suite of peptide hormone mimicry effectors, CLE, CEP, PSY, RALF, and IDA, that structurally and functionally resemble host endogenous signaling peptides. These effectors are translocated through the stylet into the plant host and interact with host receptor-like kinases, thereby hijacking developmental and immune signaling pathways to promote the establishment of feeding sites. The inset on the upper left illustrates the trafficking of CLE peptides through the ER-Golgi secretory pathway, highlighting post-translational modifications such as glycosylation guided by variable domains (VD-T). Innate signaling is modulated downstream of receptor activation, including JA signaling via MYC2, MAPK activation, and the generation of ROS, all of which lead to transcriptional reprogramming in the host nuclei. Functional annotation where effectors interact with specific receptors in the host apoplast is provided in the figure legend on the upper right. Abbreviations: CLE; CLAVATA3/ESR, CEP; C-terminally encoded peptide, PSY; Plant peptide containing sulfated tyrosine, RALF; Rapid alkalinization factor, IDA; Inflorescence deficiency, CLV1/CRN; CLAVATA1 and CORYNE receptor kinase complex, FER; FERONIA receptor kinase, CEPR1; CEP receptor 1, NLP7; NIN-like protein 7, HSL2/HAE; HAESA-like 2 and HAESA receptor kinases, PSY1R; PSY1 receptor kinase, KNOX TF; KNOTTED-like homeobox transcription factor, JA; Jasmonic acid, MAPK; Mitogen-activated protein kinase, MYC2; Master transcription factor in JA, ROS; Reactive oxygen species, SP; Signal peptide, VD-T; Variable domain for trafficking, NO3-; Nitrate ion

As the infection progresses and the nematode transitions to a sedentary lifestyle, SvG degenerates, while the DG becomes the primary source of effector proteins [44]. DG-derived effector plays central roles in host cell reprogramming, including the modulation of developmental signaling, suppression of immunity, and maintenance of the long-term biotrophic plant-nematode interphase [45–47]. Many of these late-stage effectors mimic endogenous plant peptides, such as CLE, CEP, IDA, and RALF, and are delivered into the host cytoplasmic or apoplastic spaces, where they engage receptor-like kinase and influence key developmental and stress-related pathways (Fig. 1) [35, 48]. Effector secretion is tightly coordinated with nematode developmental stages and host tissue responses. The transition in gland activity from SvG to DG underscores a fundamental shift in effector function, from facilitating invasion and suppression to maintaining long-term compatibility. Among the DG-derived effectors, peptide hormone mimics have gained increasing attention due to their potent ability to hijack plant signaling networks involved in growth, cell identity, and immune regulation. The regulation, processing, and intracellular trafficking of these specialized effectors are essential for revealing how nematodes control host physiology at the molecular level. These insights are critical for understanding the virulence roles of peptide mimicry effectors.

### Peptide hormone mimicry as a virulence strategy

#### CLE Mimicry: manipulating stem cell identity and meristem function

One of the earliest characterized examples of plant peptide mimicry in PPN is the secretion of CLAVATA3/EMBRYO SURROUNDING REGION-related (CLE) effectors, which provides a linkage between nematode effectors and plant peptides associated with host developmental pathways [49, 50]. This concept first emerged from motif-based database searches that revealed sequence similarities between plant CLE peptides and cyst nematode gland-secreted peptides, notably *HgCLE* from *H. glycines* [51]*.* These nematode-derived CLE mimics co-opt plant receptor-mediated signaling pathways to manipulate host cell fate, including the control of stem cell maintenance, vascular patterning, and differentiation, thereby establishing and expanding the NFS.

Plant CLE peptides are broadly classified into A-type, which promote cell differentiation in root and shoot apical meristems, and B-type, which often act as negative regulators where they inhibit the proliferation of procambial cells [52, 53]. They are encoded as prepropeptides with a characteristic structure, bearing an N-terminal signal peptide (SP), a variable domain (VD), and a C-terminal CLE domain, typically comprising a conserved 12-amino-acid motif [54, 55]. The propeptide undergoes a series of post-translational modifications that cleave the SP and enhance the binding ability of the CLE peptide to its target receptor. Upon cleavage of the SP, these peptides transit to the apoplast and are identified by leucine-rich repeat receptor-like kinases (LRR-RLKs), such as CLAVATA1 (clv!), triggering downstream developmental responses [56].

Similarly, CNs secrete CLE-like peptides from their dorsal gland cells, particularly during sedentary stages [57, 58]. These effectors mimic host CLEs in both structure and function, acting as molecular ligands that bind plant RLKs to promote cellular reprogramming in the developing syncytium [49, 59]. Transcriptomic analyses have identified both single-domain and multi-domain A-type CLE-like effectors across *Heterodera* and *Globodera* species, with expression patterns correlating with meristematic activity during syncytium initiation [48, 49, 60]. Comparative studies further reveal that these CLEs represent a conserved component of the CN effectorome derived from relatively newer genetic capital and progressively refined as a biotrophic adaptation that enables precise manipulation of host developmental processes [10, 61]. Importantly, this strategy converges with the host's CLE signaling, as endogenous CLE peptides are also co-opted during nematode infection to reinforce the development of NFS. Notably, during RKN infection, endogenous CLE peptides are upregulated in infected root tissues during the early stage of nematode infection [62, 63]. CLE is specifically induced in galls and plays a key role in promoting gall formation, as shown by reduced gall formation in *cle3* mutants and increased gall development upon its expression [62, 64, 65]. These findings suggest a complex interplay where nematode-secreted CLE mimics and host-induced CLEs may converge on shared RLK-mediated signaling hubs to reinforce nematode parasitism.

B-type CLE peptides have also been confirmed in *H. glycines*, where sequence alignment studies reveal significant homology with the Tracheary Element Differentiation Inhibitory Factor (TDIF), a vascular CLE peptide involved in xylem patterning [59, 66]. This suggests that nematode CLEs may additionally promote feeding site vascularization. They also appear to integrate with auxin signaling via HD-ZIP III TFs to promote tissue differentiation [67]. Consistent with these proposed roles, functional studies have confirmed that the 12-aa CLE motif is critical for biological activity [48].

In nematodes, CLE effectors are synthesized in the esophageal gland and delivered into host root cells via the stylet. Once in planta, they bind RLKs such as CLV1, triggering developmental reprogramming that favors syncytium formation. The precision with which CLE-like effectors exploit ligand mimicry to integrate host developmental programs exemplifies the sophistication of nematode parasitism and the evolutionary success of the strategy. Building on this paradigm in strategy, nematodes have evolved additional peptide hormone mimics beyond CLEs, including C-terminally Encoded Peptides (CEPs), which target nutrient signaling and root system architecture.

#### CEP Mimicry: modulating nutrient signaling and root architecture

Following the successful characterization of CN CLE-like peptides, other plant peptide-like parasitic nematode hormones have been identified, notably the CEP family, which has been characterized in sedentary and semi-endoparasitic nematodes [35]. These are endogenous plant hormones that regulate root architecture, nitrate uptake, and long-distance signaling in response to changes in nutrient availability [68–70]. In plants, CEPs are encoded by small gene families that produce prepropeptides consisting of an N-terminal signal peptide, variable regions, and one or more conserved CEP domains [68].

Surprisingly, plant-parasitic nematodes represent the only known non-plant sources of biosynthesized CEPs. CEP-like genes were first identified in *Meloidogyne* and *Heterodera* species through transcriptomic analyses [4, 71]. These nematode-derived CEPs are expressed specifically during sedentary stages and are thought to contribute to the establishment and maintenance of the feeding site. Remarkably, this activity parallels the role of plant CEPs in regulating plant developmental processes.

One well-studied example is MhCEP11, a CEP-like peptide from *M. hapla*, exhibiting striking sequence homology to *Medicago truncatula* CEP (MtCEP1) and *Arabidopsis thaliana* CEP (AtCEP1) [72]. Functional studies have shown that overexpressing the homolog of *M. trunculata,* CEP1, in plants resulted in root cell differentiation and gall-like structures that resemble nematode-induced galls [73]. This suggests a conserved role for CEPs in cell proliferation and feeding site formation, although the specific activity of RKN CEP-like peptides in planta remains to be fully elucidated.

However, we do know that structurally, RKN-derived CEPs are short peptides of approximately 15 amino acids that feature a conserved CEP domain and an extended N-terminal signal peptide. Notably, these peptides lack a pro-domain, a feature also seen in RKN CLEs, which may facilitate direct apoplastic delivery [48]. The absence of pro-domains and variable regions may streamline processing and enable rapid interaction with host receptors, thereby increasing effector efficiency. Further comparative analysis across *Meloidogyne* species revealed slight sequence variations among CEP-like peptides, suggesting species-specific adaptation and functional divergence [72]. Interestingly, even a single substitution in amino acid composition across CEPs can alter peptide activity, indicating plasticity in host-specific virulence [74]. These findings show that CEP-like peptides have evolved independently within RKN species, which may have contributed to their ability to infect a broad range of hosts and parasitic success.

These nematode CEP-like peptides represent a striking example of molecular mimicry, hijacking nutrient signaling pathways and developmental cues to promote the establishment of feeding sites. Their simplified architecture, host receptor compatibility, and evolutionary variability highlight their importance in nematode parasitism and adaptive success. By targeting nitrate signaling and lateral root development, CEP-like effectors illustrate how nematodes manipulate plant resource allocation to sustain parasitism. Remarkably, nematodes are also capable of other forms of host manipulation, such as interfering with cell separation and remodeling processes for NFS expansion by exploiting INFLORESCENCE DEFICIENT IN ABSCISSION (IDA)-like peptides.

#### IDA Mimicry: exploiting cell separation pathways for feeding site development

Another level of molecular mimicry in sedentary nematodes involves IDA-like peptides, which resemble the INFLORESCENCE DEFICIENT IN ABSCISSION (IDA) proteins of plants. In plants, IDA peptides regulate cell separation processes such as floral organ shedding and lateral root emergence by activating receptor-like kinases (RLKs) within specialized abscission zone (AZ) cells [75, 76]. These processes rely on tightly controlled cell wall remodeling that is modulated by genes upregulated during abscission, as revealed by RNA sequencing analysis [77].

The plant IDA gene encodes a small secreted peptide with an N-terminal signal peptide, a variable region, a PIP (pattern-induced peptide) motif, and a conserved C-terminal domain. This peptide binds to RLKs such as HAESA (HAE) and HAESA-LIKE2 (HSL2), triggering downstream signaling cascades that alter the KNOX transcription factor (TF) and promote organ separation [78]. Given the overlap between cell separation during abscission and cell wall remodeling during nematode feeding site formation, IDA-like signaling mechanisms have attracted interest in the context of nematode parasitism.

Initial investigation in soybean cyst nematode (SCN) suggested that IDA-mediated signaling might be co-opted during syncytium development. However, expression profiling revealed no significant upregulation of IDA-like genes in soybean roots during SCN infection [31], and genomic analyses failed to identify canonical IDA-like genes in *H. glycines*. This seemed to disregard the notion of a possible role for IDA mimicry in SCN virulence.

Surprisingly, IDA-like peptides were later identified in RKN [31]. Specifically, two RKN genes, MilDL1 and MilDL2, encode peptides with IDA-like motifs and are expressed in *M. incognita* during its sedentary stages [79]. Their discovery ignited interest in IDA mimicry as a potential virulence strategy. Notably, these peptides exhibit structural similarities to HgCLE2, a well-studied cyst nematode CLE-like effector. They can thus be predicted to be delivered apoplastically, which is also consistent with the delivery mode of many RKN effectors during the formation of giant cells.

Functional validation of IDA mimicry came from heterogeneous expression studies in *Arabidopsis*. When MilDL1 was expressed in the *ida* mutant, it sustained the floral organ abscission defect, demonstrating functional mimicry of endogenous IDA [79, 80]. Furthermore, silencing of MilDL1 genes in nematodes reduces infection, while overexpression in host plants promotes susceptibility, implying a vital role for these peptides in nematode virulence. Co-expression assays also confirmed that MilDL1 interacts with *Arabidopsis* IDA receptor-like kinases, reinforcing its role in modulating host signaling [79].

These findings support a model in which RKNs exploit IDA-like signaling to promote localized cell separation and wall loosening that initiate feeding site formation. This mechanism complements CWDE-based degradation and reflects a sophisticated adaptation of endogenous host signaling systems to benefit nematode parasitism. In a related manner, peptide-like effectors exploit host pH and cell wall signaling to provide other parasitism modes involving the modulation of developmental and defense systems.

#### RALF Mimicry: manipulating cell wall integrity and immune signaling

Among the most compelling examples of host receptor ligand mimicry in RKN is the secretion of peptides that resemble rapid alkalinization factor (RALF) proteins. RALFs are ubiquitous plant signaling peptides that regulate cell expansion, pH homeostasis, cell wall modification, and stress responses [81, 82]. They were originally identified in tobacco and characterized for their ability to induce rapid alkalinization in plant cell suspension culture [83]. Since then, its studies continue and have been established as a plant development regulator factor [84]. Subsequently, multiple studies have demonstrated their roles in diverse processes, including hormone signaling [85], cytoplasmic calcium dynamics [86], cell wall remodeling [87], and tolerance to abiotic stress [88, 89].

Plant RALF peptides are synthesized as preproproteins consisting of an N-terminal signal peptide, a variable prodomain, and a conserved C-terminal RALF domain of approximately 54 amino acids. The functional RALF domain contains two distinctive motifs, the N-terminal YISY motif for alkalinization activity and the C-terminal RGC(5 N)C motif involved in receptor interaction [90]. This suggests that the mature RALF can interact at both poles, allowing it to form various complexes via different structural changes. The maturation of RALF peptides typically involves proteolytic cleavage at a dibasic site (RRXL) upstream of the active RALF domain, releasing the functional peptide for secretion into the apoplast [91, 92].

Recent studies have shown that RKNs possess their own RALF-like peptides, capable of mimicking host RALF function and interacting with specific receptor-like kinases (RLKs) to promote parasitism [33]. The best-characterized of these is MiRALF1, secreted by *M. incognita*, which targets FERONIA (FER), a malectin-like RLK involved in sensing cell wall integrity and regulating immune responses [93]. This interaction, illustrated in Fig. 1, highlights the apoplastic engagement of mimicry effectors. In *Arabidopsis*, RKN development is significantly impaired in *fer* mutants, highlighting FERs' importance in nematode virulence [33].

A search of the *M. incognita* genome revealed 18 RALF-like peptides, many of which share conserved features with plant RALFs, including the YISY and RGC(5n)C motifs [94]. However, RKN-derived RALFs also exhibit distinct differences. Often, they lack a pro-domain, lack the canonical dibasic cleavage site and lack two cystine residues essential for disulfide bond formation in plant RALFS [90]. Experimental evidence has indicated that the pro domain is not strictly required for RALF activities, which may be the reason why they are not conserved during nematode evolution [33]. When present, nematode pro domains are shorter and structurally distinct from their plant counterparts [35]. These modifications likely reflect functional adaptation where the absence of a pro domain may facilitate more rapid secretion of the mature peptide and allow direct release of the bioactive form into the apoplast, bypassing host-like maturation steps. This streamlining of the secretion process could enhance infection efficiency by ensuring immediate interaction with host FERONIA receptors upon delivery.

However, despite these differences, nematode RALFs retain functional activity. MiRALF1, for instance, induces alkalinization in an FER-dependent manner, mirroring the function of plant RALF1 and contributing to cell wall loosening and the development of feeding sites [33, 95]. In soybean, another RALF-like peptide was shown to interact with GmLMM1, an FER-like RLK. Notably, the Gmlmm1 mutant line displayed increased resistance to RKN infection, and CRISPR-Cas9 mutagenesis was explored to confirm its role as a susceptibility gene. Additionally, a co-immunoprecipitation assay further validated the interaction between MiRALF and GmLMM1 in plants [93].

These findings highlight the refinement, over time, of RALF mimicry in RKN, where nematodes co-opt plant cell wall signaling and immune regulators to create a permissive environment for NFS development.

#### PSY Mimicry: exploiting sulfated peptide signaling for host manipulation

A more recently discovered strategy in sedentary nematode parasitism capacity is the mimicry of plant peptides containing sulfated tyrosine (PSY), a family of small, post-translationally modified peptides essential for regulating cell proliferation, elongation, and development. Originally identified in plants as phytosulfokines (PSKs), a class of 5-amino acid peptides with two tyrosine sulfates, PSY-family peptides are now recognized as critical regulators of root architecture, stomatal opening, and immune signaling [96–98]. These peptides are synthesized as prepropeptides, with an N-terminal signal peptide, a prodomain, and a short active domain. A key feature of PSY bioactivity is the sulfation of tyrosine residues, a modification catalyzed by tyrosylprotein sulfotransferases (TPSTs) [99]. The PSY1 signaling pathway is mediated by the PSY1 receptor (PSY1R), an LRR-RLK that activates downstream responses, including the plasma membrane H + -ATPase, which is required for cell expansion and pH regulation [100, 101].

Interestingly, PSY-like peptides have also been identified in RKN also, notably, *M. incognita*, where they are proposed to act as molecular mimics of endogenous PSYs. This builds upon previous discoveries of sulfated peptide mimics in other pathogens, such as the RaxX peptide of *Xanthomonas oryzae*, which binds to the rice XA21 receptor and modulates host immunity [102]. The identification of such peptides in PPN highlights the convergent evolution of biotrophic parasites towards exploiting host peptide signaling mechanisms.

In RKNs, PSY-like peptides such as MigPSYs have been identified as 50-aa prepropeptides containing an N-terminal secretion signal and a conserved PSY domain. A distinctive feature of MigPSY is the presence of a conserved tyrosine motif downstream of an aspartic acid, supporting the likelihood of tyrosine sulfation, which is essential for peptide activity [32]. Unlike bacterial or plant PSYs, MIgPSYs lack a variable domain, which may facilitate direct delivery into the apoplast, bypassing complex intracellular processing and enabling rapid interaction with host receptors.

Comparative genomics has shown that PSY peptide genes are conserved in clade 1 *Meloidogyne* species but absent in other clades [32], suggesting clade-specific evolutionary acquisition. Specifically, MigPSY is classified into three variants (MigPSY1, MigPSY2, and MigPSY3) based on conserved domain differences. Notably, MigPSY1 occurs in *M*. *arenaria*, MigPSY2 in both *M. arenaria* and *M. javanica* but not in *M. incognita.* MigPSY3, distinguished by a 2-amino-acid SR insertion between the DY and NXXHXP motifs, is the most widely represented, with approximately three genomic copies in each clade I species. Interestingly, PSY-like motifs are also encountered in the genomes of other clade 1 *Meloidogyne* species, including *M. enterolobii*, *M. floridensis*, and *M. luci* [32]. The strong conservation of MigPSY3 across clade 1 RKN likely reflects its adaptive role in supporting broad host compatibility. These species, including *M. incognita*, *M. javanica*, and *M. arenaria*, are highly polyphagous and are known to infect numerous plant taxa. The SR insertion and domain variability in MigPSY3 may confer receptor-binding flexibility, allowing manipulation of PSY-dependent signaling across diverse hosts. This functional versatility may be due to its retention as the dominant PSY mimic within Clade 1 and underscores its contribution to their remarkable parasitic success. This clade-restricted distribution, combined with the structural simplicity of MigPSYs, points to their potential significance in adaptive success and broad host range.

Functional analysis of MigPSYs has provided compelling evidence for their role in parasitism. Further silencing of PSY-like genes in *M. incognita* impairs both nematode development and NFS formation in rice [32], supporting its role in the infection process. Thus, mechanistically, these peptides act analogously to plant PSYs in promoting key components of gall formation, such as cell expansion and proliferation. Their sulfated nature and apoplastic emergence further support a model where post-translational modification and precise delivery are critical for virulence. In essence, PSY-like peptides in RKN exemplify a sophisticated virulence strategy, mimicking plant sulfated peptide hormones to manipulate host development and immunity. Collectively, their evolutionary convergence with other pathogens reflects the importance of plant peptide mimicry as a core mode in parasitism. However, the success of these mimicry strategies largely depends on how the effectors are synthesized, processed, and delivered to the host cellular components.

## Trafficking, processing, and host targeting of peptide effectors

The biological capacity of nematode-derived peptide effectors relies not only on their structural mimicry of host peptides but also on their precise biosynthesis, post-translational modifications, and trafficking that ensure timely delivery to their target subcellular compartment. These sophisticated regulatory periods are critical for stabilizing effectors in the plant's hostile extracellular environment and for enabling effective interaction with specific host receptors.

Peptide effectors are synthesized in the nematode’s esophageal gland as prepropeptides, which typically contain an N-terminal signal peptide that directs them into the secretory pathway. Upon entry into the endoplasmic reticulum, the signal peptide is cleaved, allowing proper synthesis and subsequent processing. Many nematode prepropeptides contain internal processing motifs, which are recognized by nematode-encoded proteases to release mature, active peptides. This proteolytic processing is crucial, ensuring that only the functional form of the peptide is ultimately delivered into host tissues.

For instance, *GrCLE1* from *G. rostochiensis* is processed into a 12-amino acid glycopeptide, GrCLE1-1Hyp4,7 g, which contains hydroxylated prolines and a tri-arabinosylated Hyp at position 7, enhancing receptor affinity and activity [103]. Similarly, the *MhCEP11* peptide from *M. hapla* requires hydroxylation at P4 and P11 for full functionality [73]. Interestingly, some peptides deviate from these canonical processing pathways. For example, RALF peptides lack the classical cleavage motifs and pro domains, instead relying on alternative or host-assisted mechanisms for activation (see RALF Mimicry section) [33]. PSY-like peptides are cleaved just after their N-terminal signal sequence to release a short active motif-containing peptide.

## The crucial role of post-translational modifications (PTMs) in Effector Functionality

Post-translational modifications play a vital role in enhancing effector stability and ensuring high-affinity receptor interactions, which are crucial for functional mimicry. In CLE-like effectors, proline hydroxylation and tri-arabinosylation are essential for receptor engagement, especially plant RLKs such as CLV1 [103, 104]. These sugar additions also protect peptides from proteolytic degradation, particularly in the apoplast. In vitro assays have confirmed that arabinosylated GrCLEs resist host proteases more effectively than their non-modified counterparts [103]. Incomplete modification yields intermediate forms such as GrCLE1-1Hyp7 and GrCLE1-1Hyp4, 7, which show reduced activity, further confirming the functional importance of full PTMs during parasitism.

Similarly, the importance of PTMs is evident in PSY-like effectors, where tyrosine sulfation, likely mediated by a nematode-encoded sulfotransferase, enhances the mimicry of host PSYs and facilitates their interaction with PSY1 receptors [32, 99]. Pathogens such as *Xanthomonas oryzae* exploit similar strategies, emphasizing the evolutionary convergence of virulence mechanisms [102]. CEP-like peptides also undergo hydroxylation and arabinosylation, which ensures their interaction with host receptors, as peptides deprived of these PTMs exhibit residual or no activity [73, 105].

## Effector trafficking and host target engagement

The delivery routes of peptide effectors also have crucial roles in defining their activity. Some effectors are secreted directly into the apoplast, where they interact with extracellular domains of host RLKs to trigger specific downstream signaling cascades. A prime example is the RALF-like peptides secreted by *M. incognita*, which bind to the extracellular domain of the FER receptor in the apoplast, thereby modulating cell wall integrity and immune signaling [33, 93]. Similarly, MilDL1, an IDA-like effector, also functions via apoplastic targeting [79].

CEP-like and PSY-like peptides are thought to follow a similar secretion route. Their lack of a variable domain, combined with a strong N-terminal signal peptide, supports their direct apoplastic delivery [35, 106]. While some PTMs likely occur within the nematode gut, host-based processing cannot be ruled out. In contrast, other effectors, such as cyst nematode HgCLE2, are first delivered into the host cytoplasm, after which they are trafficked to distal apoplastic targets [48]. This intracellular route, influenced by the N-terminal variable region, allows these effectors to engage more complex developmental processes within the host tissue.

Beyond the specific processing and trafficking routes, the spatial and temporal regulation of effector secretion is paramount to successful parasitism. Notably, the peptide ligand requires precise regulation to facilitate the targeted modulation of plant developmental processes [107]. Most peptide effectors involved in NFS formation are specifically expressed in the dorsal gland, particularly during sedentary periods [108]. This stage-specific regulation ensures that effectors are secreted during NFS initiation and maintained during its development.

Additionally, some nematode effectors appear to synchronize with host developmental cues, potentially exploiting periods of maximal receptor availability or host vulnerability to optimize the biotrophic interaction. For example, CLE effectors in *H. glycines* are highly expressed during host root meristem activity, which enables the alignment of nematode manipulation with critical stages of plant growth [49].

Collectively, these molecular features, from prepropeptide synthesis and proteolytic cleavage to precise PTMs, trafficking routes, and precise spatial and temporal regulation, form an integrated regulatory system that profoundly modulates the virulence capacity of nematode peptide effectors. A deeper understanding of these intricate layers of regulation will be crucial for designing next-generation strategies, such as engineering transgenic crops that disrupt effector enhancement or receptor recognition, offering novel avenues to durable nematode resistance.

## Evolutionary emergence of mimicry effectors

The functional sophistication of nematode peptide effectors, particularly their role in virulence through structural and functional resemblance to plant signaling molecules, raises fundamental questions about their evolutionary origin. As discussed in the previous sections, the bioactivity of mimicry effectors depends not only on structural similarity to host peptides but also on specialized processing and delivery mechanisms. This complexity implies that their emergence involved more than gradual divergence. Instead, it reflects a convergence of multiple evolutionary processes, including cross-kingdom mimicry, horizontal gene transfer (HGT), gene duplication and neofunctionalization, and adaptive sequence evolution [19, 109, 110].

Taking on a major route of innovation, cross-kingdom mimicry represents a major evolutionary innovation in nematodes, enabling effectors to structurally and functionally resemble plant peptides such as CLE, CEP, IDA, PSY, and RALF peptides. This molecular deception provides a direct pathway for RKN to hijack host regulatory networks through conserved structural replications. For instance, sequence comparisons have revealed that nematode mimics retain conserved core motifs (e.g., RLVPRGPDPIHN in CLEs, DY sulfation site in PSYs) [32, 111] and incorporate short lead peptides resembling plant signal sequences to ensure apoplastic secretion. These molecules rely on similar PTMs and engage specific host RLKs to directly integrate into plant developmental and signaling networks [35].

**Fig. 2:**
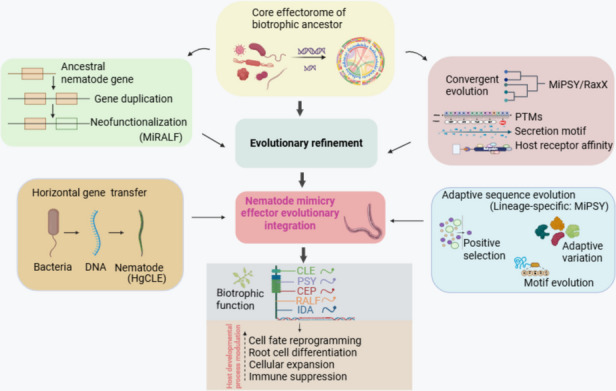
Evolutionary Pathways Leading to the Emergence of Peptide Hormone Mimicry Effectors in Sedentary Plant-Parasitic Nematodes. There are multifaceted evolutionary processes underlying the origin and refinement of peptide mimicry effectors. The core effectorome of a common biotrophic ancestor suggests a genetic pool from which recent annotated effector genes evolved. Key factors include gene duplication and neofunctionalization of acquired nematode genes. Horizontal gene transfer from other organisms contributes foreign genetic elements. There is also the acquisition of secretion motifs, post-translational modification, and host receptor affinity, converging to augment the peptide hormone repertoire. Adaptive sequence evolution is marked by positive selection, motif diversification, and structural imitation. These factors, through evolutionary refinement, produce mimicry effector integration, enabling functional specialization of CEL, CEP, RELAF, PSY, and IDA peptide mimics

Interestingly, comparable strategies have evolved independently in other pathogens, such as the RaxX peptide of *Xanthomonas oryzae*, which mimics plant PSY peptides to bind the XA21 receptor and modulate rice immunity. This parallel evolution between bacteria and nematodes highlights convergent evolutionary pressure to exploit conserved peptide-receptor modules as molecular entry points into host signaling. In nematodes, such mimicry traits likely emerged through the repurposing of ancestral secretory genes, refined by neofunctionalization and host-driven selection to optimize trafficking and receptor affinity. Together, these findings demonstrate how convergent evolution has repeatedly produced biotrophic strategies centered on peptide hormone mimicry, reinforcing it as a hallmark of sedentary nematode parasitism (Fig. 2).

Horizontal gene transfer has also played a pivotal role in expanding effector repertoires by providing the raw genetic material for functional innovation. Nematodes have acquired numerous virulence-related genes from bacteria, fungi, or even their plant host [112, 113]. This process is particularly significant for peptide effectors because it introduces novel sequences that can be repeatedly adapted. For example, certain CLE-like genes in cyst nematodes lack clear phylogenetic continuity within the nematode lineage. They may have originated from rhizosphere microbes [114], suggesting that HGT provided the foundational peptide scaffold for mimicry. Similar evidence has been reported in *Globodera rostochiensis*, where bacterial-derived cell wall-degrading enzymes were horizontally transferred and subsequently repurposed for nematode parasitism [115], exemplifying how such events can yield functional adaptation in nematodes. Comparative genomics further supports this pattern. Approximately 54% of effector families in *H. schachtii* show no detectable homology outside sedentary nematodes [61], indicating either recent acquisition or rapid post-HGT divergence driven by host-imposed selection. The frequent presence of transposable elements near effector loci [113, 116] may facilitate the shuffling of promoter regions, signal peptides, and secretion motifs, enhancing functional adaptation. Thus, while HGT is not a mimicry mechanism itself, it represents a crucial precursor that introduces foreign genes later refined through duplication and selection to structurally and functionally mimic host peptides.

Gene duplication and neofunctionalization subsequently expand and refine this effector repertoire. In *H. schachtii,* approximately 19% of effectors, representing 11% of effector families, share homology with highly conserved eukaryotic genes, suggesting their emergence via duplication and functional repurposing of ancestral secretory genes into new virulence signals [61]. Duplication events may have expanded effector copy numbers to diversify variable domains, which now modulate trafficking and receptor-binding specificity. For instance, duplicated CLE homologs differ in their N-terminal signal peptides, influencing secretion routes and host receptor affinity while maintaining a conserved C-terminal CLE motif responsible for biological activity [80]. Such diversification has enabled nematodes to regulate secretion timing and host recognition, facilitating adaptation to diverse plant signaling cues.

Comparative genomics further reveals that migratory nematodes rely on peptidases and pectin-degrading enzymes for mechanical invasion and transient nutrient extraction [112, 117]. Conversely, sedentary species have evolved a distinct strategy, shifting towards effectors that mimic plant developmental signals [118, 119]. This shift necessitated the evolution of signals that enable long-term modulation of host transcriptional programmes essential for NFS development. This evolutionary trade-off between mechanical parasitism and molecular manipulation underscores how sedentary nematodes refine their effectoromes for sustained compatibility with host tissues.

Adaptive sequence evolution further diversifies effector families. Many effectors evolve under positive selection, especially at host interaction residues [112], which fine-tune mimicry motifs for receptor affinity and optimize PTM-associated regions for efficient modification. Their modular design, comprising signal peptides, internal cleavage motifs, and a conserved core, allows for adaptive variation within mimicry domains without disrupting secretion and activation mechanisms. This modularity accelerates the evolution of highly specialized mimics, as seen in approximately 6% of *H. schachtii* effectors that are lineage-specific and uncharacterized, lacking homologs even in other species, including the closely related *H. glycines* [61]. These lineage-specific molecules likely represent recent host-adaptative innovations refined to target distinct receptors. Notably, the repeated evolution of structurally distinct effectors converging on the same plant peptide targets underscores the strong selective advantage of hijacking conserved RLK signaling modules, regardless of molecular origin.

Taken together, cross-kingdom mimicry, HGT, neofunctionalization, and adaptive evolution converge to shape the emergence of peptide mimicry effectors in sedentary nematodes. These dynamic and interlinked processes have produced a specialized effector repertoire capable of hijacking plant developmental and immune systems with remarkable specificity. Tracing the origins and diversification of these effector genes enhances our understanding of plant-nematode coevolution and identifies molecular targets for resistance breeding and biotechnological control.

### Future directions and research gaps

Despite major advances in the discovery and functional annotation of nematode effectors, particularly peptide hormone mimics, several key questions remain unresolved in fully understanding their roles in parasitism. More in-depth research on how these effectors are synthesized, processed, trafficked, and ultimately perceived and activated within host tissues is central to unlocking novel strategies for nematode control. Hence, there is a need to highlight critical gaps in current knowledge and propose future research areas that bridge fundamental biology with translational approaches for plant protection.

#### Decoding effector perception and specificity in host tissues

One of the compelling gaps involves how plant receptor systems differentiate between nematode-derived peptide mimics and their endogenous counterparts. While nematode CLEs, CEPs, IDAs, RALFs, and PSYs often bind the same or analogous receptor-like kinases (RLKs) as plant peptides, the degree of mimicry specificity, competition, or antagonism remains poorly understood. A key question is whether these nematode peptides competitively displace endogenous peptides or if they subtly reprogram them towards parasitism. Interestingly, plant species are capable of deploying R proteins that recognize nematode molecules [114, 120], but it appears that they are unable to distinguish between mimicry and authentic endogenous signaling. This suggests that mimicry can escape immune responses despite direct interaction with the effector molecule.

Recent advances in structure-based prediction now offer promising tools to explore these interfaces computationally. A recent study on *Bursaphelenchus xylophilus* applied a deep-learning model where they predicted protein interactions between the pine wood nematode and pine using deep learning and multi-dimensional feature fusion that integrated AlphaFold-derived 3D features [121]. Such approaches could be adapted to model effector-receptor complexes in sedentary nematodes, allowing identification of potential interface residues and contact domains. These computational insights, coupled with experimental validation through cryo-EM or X-ray crystallography [122], could reveal the structural basis of receptor recognition and specificity in peptide mimicry. Ultimately, this could enhance the understanding of downstream signaling and the receptor activation milieu, leading to the engineering of plants capable of isolating mimicry without disrupting innate peptide signaling pathways.

#### Trafficking, post-translational modification, and in planta activation

Another significant gap lies in the intracellular trafficking and biochemical maturation of peptide effectors both within the nematode and in the host tissues. While some notable effectors undergo initial processing within the nematode esophageal gland, compelling evidence has shown that several others rely on host machinery for full maturation [123, 124]. They utilize host factors for cleavage, hydroxylation, arabinosylation, or sulfation, all of which are essential for full functionality. However, the full annotation, origin, and temporal regulation of active enzymes mediating the PTM remain poorly understood. Similarly, emerging insights have highlighted the role of the ER not only in effector trafficking but also as a battleground in plant host‒pathogen interactions [125]. Thus, it is necessary to note the functional homeostasis of the ER, which is crucial for the survival of plant cells, and how the host can trigger ER stress responses to repress nematode effector proteins.

Future research should prioritize mapping the PTM landscape of peptide effectors using advanced proteomics. This will enable precise identification of modification sites and the stoichiometry of the modification types [126]. For example, while the current understanding of CLE trafficking confirms that the VDI signal guides trafficking through the ER secretory pathway, we do not know the specific types of PTMs or their precise modification site. This is a significant gap that advanced proteomics could fill.

Additionally, live-cell imaging using smaller fluorescent reporters holds promise for visualizing effector dynamics in real time. While fusing large tags (e.g., GFP/mCherry fusions) to VDI-containing effectors has been shown to disrupt trafficking [48], novel split-fluorescent systems or minimal peptide tags may enable unrestricted secretion. These could help visualize the differential localization of effectors to cellular spaces, providing critical insight into how contextual trafficking mediates host‒pathogen signaling and parasitism. Complementarily, proximity-labeling techniques such as TurboID and BioID offer powerful means to identify host proteins that physically interact with effectors during trafficking and PTM. The approach has been successfully explored in animal cells, plants, yeast, and fungi [127, 128]. By mapping these temporal interactions, such tools could uncover the host machinery co-opted by nematodes for effector modification and delivery.

#### Evolutionary pathways and functional divergence

Although HGT, gene duplication, and adaptive evolution have been implicated in the emergence of peptide mimicry, the timing and lineage-specific diversification of these effectors remain speculative. Moreover, the current plethora of functional studies has relied on short-term expression assays, which do not always capture the layered dynamic signaling reprogramming that underlies nematode parasitism. Moreover, the interaction among multiple effectors over time and space is rarely considered. In this regard, future research should intensify comparative genomics and phylogenomic analysis across a wide range of sedentary and migratory nematodes. Currently, such studies are mostly limited to a particular PPN feeding category [129, 130]. Expanding the study will provide a robust reconstruction of effector phylogenies and insights into how specific effector clades emerged or diversified in parallel with their biotrophic lifestyle. Moreover, evolutionary rate analyses, such as dN/dS ratios, can also reveal residues under strong selection, particularly within the mimicry domain and receptor-interacting motifs [109]. These studies will deepen our understanding of the molecular arms race between nematodes and their host, therefore revealing how parasitism strategies have evolved to co-opt plant signaling systems.

#### Translational and biotechnological applications

Integrating effector biology into plant protection frameworks offers a promising avenue for sustainable nematode control. Notably, available breeding tools such as CRISPR/Cas9 could be used to modify host RLKs to evade effector binding while maintaining normal signaling. Similarly, host-induced gene silencing (HIGS) and RNA interference (RNAi) targeting key effectors or their modifying enzymes can offer targeted control of nematode infection [131]. This may be plausible for effector-based diagnosis, which can allow early detection of nematode presence before symptoms appear. Moreover, recent advances in synthetic biology have enabled the construction of decoy receptors or synthetic peptides that competitively inhibit effector function [132, 133]. In essence, developing plants that express such constructs may enable interception or sequestration of effectors before they reach their target.

Together, these directions underscore the dynamic and evolving field of nematode effector biology. After all, as our molecular and evolutionary understanding deepens, so does the potential for translating these insights into innovative and durable strategies for managing nematode parasitism.

## Conclusion

The study of peptide hormone mimicry in sedentary plant-parasitic nematodes has unveiled a highly evolved molecular strategy by which these organisms hijack host signaling pathways to establish and maintain long-term feeding structures. Through structural mimicry, post-translational modifications, precise trafficking, and receptor engagement, nematode effectors remarkably emulate endogenous plant peptides. This mimicry underscores the sophistication of nematode parasitism and highlights the vulnerability of conserved plant developmental networks to biotrophic exploitation.

Despite growing insights into the structure and function of peptide-like effectors, many aspects of their synthesis, host perception, and in planta dynamics remain poorly characterized. The complexity of these interactions, spanning cellular compartments, developmental stages, and evolutionary timescales, demands a more integrated research approach. High-resolution structural biology, single-cell transcriptomics, advanced proteomics, and comparative genomics will be instrumental in dissecting these molecular dialogues at a fine scale.

Equally important is the translation of this knowledge into practical resistance strategies. Genome editing, synthetic biology, effector-based diagnostics, and targeted gene silencing represent promising technologies for disrupting effector function or blocking host susceptibility. However, successfully applying these advancements will require a systems-level understanding of how nematodes coordinate effector deployment across space and time and how plants integrate or resist these signals at the network level.

Ultimately, advancing our understanding of peptide effector biology will not only deepen our grasp of plant-nematode coevolution but also equip us with molecular tools to engineer crops with durable, broad-spectrum resistance. In an era where sustainable agriculture is critical, targeting the mimicry interface offers a compelling avenue for nematode control rooted in molecular precision and ecological compatibility.

## Data Availability

Not applicable.
